# Capture Sequencing to Explore and Map Rare Casein Variants in Goats

**DOI:** 10.3389/fgene.2021.620253

**Published:** 2021-02-23

**Authors:** Siham A. Rahmatalla, Danny Arends, Ammar Said Ahmed, Lubna M. A. Hassan, Stefan Krebs, Monika Reissmann, Gudrun A. Brockmann

**Affiliations:** ^1^Animal Breeding Biology and Molecular Genetics, Albrecht Daniel Thaer-Institute of Agricultural and Horticultural Sciences, Humboldt University of Berlin, Berlin, Germany; ^2^Department of Dairy Production, Faculty of Animal Production, University of Khartoum, Khartoum North, Sudan; ^3^Animal Resource Research Corporation, Wildlife Research Center, Federal Ministry of Livestock, Fisheries and Rangelands, Khartoum North, Sudan; ^4^Labor für Funktionelle Genomanalyse, Genzentrum, Ludwig-Maximilians-Universität (LMU), Munich, Germany

**Keywords:** casein, polymorphism, genetic variation, Sudanese goats, Saanen, Bezoar ibex, Nubian ibex, Alpine ibex

## Abstract

Genetic variations in the four casein genes *CSN1S1*, *CSN2*, *CSN1S2*, and *CSN3* have obtained substantial attention since they affect the milk protein yield, milk composition, cheese processing properties, and digestibility as well as tolerance in human nutrition. Furthermore, milk protein variants are used for breed characterization, biodiversity, and phylogenetic studies. The current study aimed at the identification of casein protein variants in five domestic goat breeds from Sudan (Nubian, Desert, Nilotic, Taggar, and Saanen) and three wild goat species [*Capra aegagrus aegagrus* (Bezoar ibex), *Capra nubiana* (Nubian ibex), and *Capra ibex* (Alpine ibex)]. High-density capture sequencing of 33 goats identified in total 22 non-synonymous and 13 synonymous single nucleotide polymorphisms (SNPs), of which nine non-synonymous and seven synonymous SNPs are new. In the *CSN1S1* gene, the new non-synonymous SNP ss7213522403 segregated in Alpine ibex. In the *CSN2* gene, the new non-synonymous SNPs ss7213522526, ss7213522558, and ss7213522487 were found exclusively in Nubian and Alpine ibex. In the *CSN1S2* gene, the new non-synonymous SNPs ss7213522477, ss7213522549, and ss7213522575 were found in Nubian ibex only. In the *CSN3* gene, the non-synonymous SNPs ss7213522604 and ss7213522610 were found in Alpine ibex. The identified DNA sequence variants led to the detection of nine new casein protein variants. New variants were detected for alpha S1 casein in Saanen goats (*CSN1S1*^∗^C1), Bezoar ibex (*CSN1S1*^∗^J), and Alpine ibex (*CSN1S1*^∗^K), for beta and kappa caseins in Alpine ibex (*CSN2*^∗^F and *CSN3*^∗^X), and for alpha S2 casein in all domesticated and wild goats (*CSN1S2*^∗^H), in Nubian and Desert goats (*CSN1S2*^∗^I), or in Nubian ibex only (*CSN1S2*^∗^J and *CSN1S2*^∗^K). The results show that most novel SNPs and protein variants occur in the critically endangered Nubian ibex. This highlights the importance of the preservation of this endangered breed. Furthermore, we suggest validating and further characterizing the new casein protein variants.

## Introduction

Goats play an essential role in rural areas of developing countries. They provide food security, preservation of basic nutrition, and economic income (Dubeuf et al., 2004; Peacock, 2005; Devendra and Liang, 2012). Furthermore, goat’s milk is an essential contribution to human nutrition, especially for people who are lactose-intolerant or sensitive to cow’s milk. Goat’s milk has been associated with low allergenic reactivity, antioxidant and anti-inflammatory effects, and prevention of atherosclerosis and cardiovascular diseases (Haenlein, 2004; O’Shea et al., 2004; Russell et al., 2011; Lad et al., 2017).

Milk composition influences its nutritional value, technological properties, and the quality of dairy products (Yasmin et al., 2012). The protein fraction of goat’s milk, similarly to other ruminants, consists of casein and whey proteins (Fox et al., 2000). In goats, the caseins make up about 80% of milk proteins (Martin et al., 2002; Hayes et al., 2006). Alpha S1 (αs1-CN), beta (β-CN), and alpha S2 (αs2-CN) are calcium-sensitive caseins. Kappa casein (κ-CN) is essential for stabilizing the casein micelles (Alexander et al., 1988). Due to the specific casein protein structures, goat’s milk contains larger micelles, including more calcium and other minerals, compared to cow’s milk. The high contents of calcium and other minerals as well as the larger casein micelles improve the cheese-making properties of goat’s milk (Park et al., 2007).

The genes corresponding to alpha S1, beta, alpha S2, and kappa caseins are *CSN1S1*, *CSN2*, *CSN1S2*, and *CSN3*, respectively. The casein genes are located in a 250-kb region between 85,978 and 86,211 Mb on *Capra hircus* chromosome 6 in the following order: *CSN1S1* (85,978–85,995 Mb), *CSN2* (86,006–86,015 Mb), *CSN1S2* (86,077–86,094 Mb), and *CSN3* (86,197–86,211 Mb) (Martin et al., 2002; Cosenza et al., 2005). Casein genes appear to be rapidly evolving (Prinzenberg et al., 2005). They are highly polymorphic in all species. In *C. hircus*, 19 protein variants for alpha S1 casein (A, A2, A3, B1, B2, B3, B4, C, D, E, F, G, H, I, L, M, N, 01, and 02), eight for beta casein (A, B, C, D, E, 0, 0′, and another unnamed variant), 10 for alpha S2 casein (A, B, C, D, E, F, G, 0, and truncated sub-variants A and E), and 23 for kappa casein (A, B, C, D, E, F, G, H, I, J, K, L, M, N, O, P, Q, R, S, T, U, V, and W) have been reported (Table 1). Additional sequence variation was found in the upstream and downstream gene regions, which is hypothesized to affect the expression of the casein genes and influence the amount and ratio of the different caseins in goat’s milk (Cosenza et al., 2007; Najafi et al., 2014).

**TABLE 1:** Known variants of casein proteins.

**Protein**	**Gene**	**Protein variants**	**References**
α_*s1*_-CN	*CSN1S1*	A, A_2_, A_3_, B_1_, B_2_, B_3_, B_4_, C, D, E, F, G, H, I, L, M, N, 01, 02	Boulanger et al., 1984; Grosclaude et al., 1987; Brignon et al., 1989, 1990; Mahé and Grosclaude, 1989; Leroux et al., 1990, 1992; Jansàpérez et al., 1994; Martin and Leroux, 1994; Chianese et al., 1997; Grosclaude and Martin, 1997; Martin et al., 1999; Ramunno et al., 2000, 2004, 2005; Bevilacqua et al., 2002; Cosenza et al., 2003, 2008; Mestawet et al., 2013
β-CN	*CSN2*	A, B, C, D, E, 0, 0′, another unnamed variant	Mahé and Grosclaude, 1993; Rando et al., 1996; Rando, 1998; Persuy et al., 1999; Neveu et al., 2002; Galliano et al., 2004; Chessa et al., 2005; Caroli et al., 2006; Chianese et al., 2007
α_*s2*_-CN	*CSN1S2*	A, B, C, D, E, F, G, 0, truncated sub-variants A and E	Boulanger et al., 1984; Bouniol, 1993; Bouniol et al., 1994; Veltri et al., 2000; Lagonigro et al., 2001; Ramunno et al., 2001a,b; Erhardt et al., 2002; Cunsolo et al., 2006
κ-CN	*CSN3*	A, B, C, D, E, F, G, H, I, J, K, L, M, N, O, P, Q, R, S, T, U, V, W	Coll et al., 1993; Caroli et al., 2001; Yahyaoui et al., 2001, 2003; Angiolillo et al., 2002; Jann et al., 2004; Prinzenberg et al., 2005; Kiplagat et al., 2010; Gautam et al., 2019

*Known variants for casein mature proteins published in recent literature.*

In this study, we assessed the genetic variation in and between five different goat breeds from Sudan and three wild species. The four major domestic goat breeds in Sudan are Nubian, Desert, Nilotic, and Taggar (Rahmatalla et al., 2017). In Sudan, Nubian dairy goats are the most widely used (Wilson, 1991; Steele, 1996). The Desert goat is a feed-efficient dual-purpose breed for milk and beef production under harsh environmental conditions (Ismail et al., 2011). The Nilotic goat is a short-statured meat breed known for its high fertility and resistance to trypanosomiasis (Osman et al., 2008). Taggar is a dwarf meat goat that is adapted to the mountainous conditions (Bushara and Abu Nikhaila, 2012). More information about the Sudanese native goat breeds can be found in Rahmatalla et al. (2017). The Saanen goats used in this study were imported to Sudan from the Netherlands. While Bezoar ibex is considered as the ancestor of current domesticated goat breeds, Nubian ibex are goats from mountainous regions in Sudan. Alpine ibex are used for comparison with Nubian ibex, which both live in high mountain areas.

High-density capture sequencing was used to identify genetic variants in the casein genes. Since we did not examine proteins, we predicted protein variants from DNA polymorphisms using bioinformatics tools. The identification of such variations on the DNA and protein levels is the first step for subsequent association studies, which will provide further information about the effects of specific protein variants on milk characteristics and offer their application for breed improvement. Moreover, the information can be used for conservation decisions and further elucidation of the evolution of *Capra*.

## Materials and Methods

### Animals and Sampling

Thirty-three unrelated female goats were chosen from different regions following the recommendation of the International Society for Animal Genetics (ISAG) and the advisory group regarding animal genetic diversity of the Food and Agriculture Organization of the United Nations (FAO, 2011), as described previously (Rahmatalla et al., 2017; Hassan et al., 2018). Nubian goats (*n* = 7) were sampled from four locations in the three states along the river Nile. Desert goats (*n* = 5) from the Bara and Abu Zabad area in the North Kordofan state, Nilotic goats (*n* = 7) from the Kosti and Rabak areas in the White Nile state, and Taggar goats (*n* = 7) from the Nuba Mountains and Dalang area in the South Kordofan state. Saanen goat samples (*n* = 2) were obtained from a goat improvement farm in Khartoum state. Blood was taken from the jugular vein through the use of vacutainer tubes containing EDTA as an anticoagulant (5 ml). DNA was extracted using the Puregene core kit A (Qiagen, Hilden, Germany). Additional DNA samples were obtained from Bezoar ibex (*n* = 2), Nubian ibex (*n* = 2), and Alpine ibex (*n* = 1). DNAs of Bezoar and Alpine ibex were obtained from the DNA and tissue bank of the Leibniz Institute for Zoo and Wildlife Research Berlin, Germany. Nubian ibex originated from the Red Sea state of Sudan, as described previously (Hassan et al., 2018). Detailed information on the samples and the number of sequenced animals is given in Supplementary Table 1. The geographical location of the Sudanese goat samples included in this study is shown in Supplementary Figure 1.

### Sequencing

The casein genes *CSN1S1*, *CSN2*, *CSN1S2*, and *CSN3* were enriched by hybridization to a custom tiling array (custom designed by e-array, repeat-masked, 1-bp tiling; Agilent 244K Capture Array, Agilent, Santa Clara, United States) and sequenced on a Hiseq1500 instrument (Illumina, San Diego, United States) in paired-end mode with a read length of 100 nt. The gene-specific tiling array was created using the goat reference sequences (version LWLT01) (Bickhart et al., 2017) available at the National Center for Biotechnology Information (NCBI)^1^. The amplified regions covered 5,000 bp before the transcription start sites and 1,000 bp after the 3′-UTR region of each casein gene.

For the generation of sequencing libraries, 500 ng of genomic DNA was sheared by sonication (Covaris M220, Covaris, Woburn, MA, United States) for 75 s (20% duty factor, 200 cycles per burst) and further processed with the Accel-DNA 1S kit (Swift Biosciences, Ann Arbor, United States) according to the manufacturer’s instructions. The resulting whole genome libraries were barcoded and pooled in equimolar amounts and hybridized to the tiling array. Briefly, the libraries were hybridized for 65 h at 65°C, washed, and eluted with nuclease-free water for 10 min at 95°C. The eluted DNA was concentrated in a vacuum centrifuge, amplified by PCR (10 cycles with 98°C for 15 s, 65°C for 30 s, and 72°C for 30 s) and purified with Ampure XP beads.

### Data Analysis

Fastq sequencing files were demultiplexed based on their barcodes and reads were trimmed using trimmomatic, after which the trimmed reads were aligned to the LWLT01 reference genome using BWA. The BAM files containing the raw aligned reads per sample formed the input for our variant calling pipeline. Variants were called using BCFtools, and the resulting raw single nucleotide polymorphism (SNP) calls were filtered using the varFilter tools from the vcfutils package to remove all off-target calls (Danecek et al., 2011).

Two settings in the SNP calling phase were adjusted: read quality (-*q*) in the mpileup step was increased to 30 (default, 0), and varFilter in the vcfutils step was called with a minimum depth (-*d*) of 10 reads (default: 2) in a sample before a SNP was to be called. All other settings for SNP filtering were left to their default values. This means that if a SNP is called in a sample, at least 10 reads with a read quality of 30 were available to support the detected SNPs. All SNPs have a minimal QUAL score in the resulting VCF file of 300. The average read depth across all SNPs was visualized and is available in Supplementary Figure 2. Polymorphisms were validated visually using the Integrative Genomics Viewer (IGV) (Robinson et al., 2011).

The positions of the identified sequence variants presented in this paper are based on the LWLT01 genome. Known sequence variants were annotated based on the Single Nucleotide Polymorphism database (dbSNP, build 143) and are reported in this paper with their rs identification number and novel SNPs with their European Variant Archive (EVA) ss identifier.

Subsequently, novel high confident SNPs were combined and annotated using a custom R script that relies on the “seqinr” and “Biostrings” R packages. In short, the GFF3 file containing the gene, exon, and cds locations for goat was combined with the SNPs from the VCF file obtained from SNP calling using R 4.0.2. Amino acid changes were determined by translating codons using the “Biostrings” package (Pagès et al., 2020) using “The Standard Genetic Code” codon table.

The effects of novel non-synonymous SNPs on protein function were predicted using the PROVEAN tool (Choi and Chan, 2015)^2^ and are available in Supplementary Table 2. The peptide chain response to hydrolysis and cleavage was tested for the amino acid sequences with mutated amino acids in casein proteins by using the PeptideCutter program at ExPASy Bioinformatics Resource Portal^3^. The isoelectric focus (IEF) information for the new protein variants in the kappa casein was not experimentally tested, but predicted using the ExPASy tool whether the variant belonged to the A^*IEF*^ or the B^*IEF*^ group in the kappa casein protein (Gasteiger et al., 2003).

## Results

### DNA Sequence Variants in the Casein Gene Regions

**FIGURE 1:**
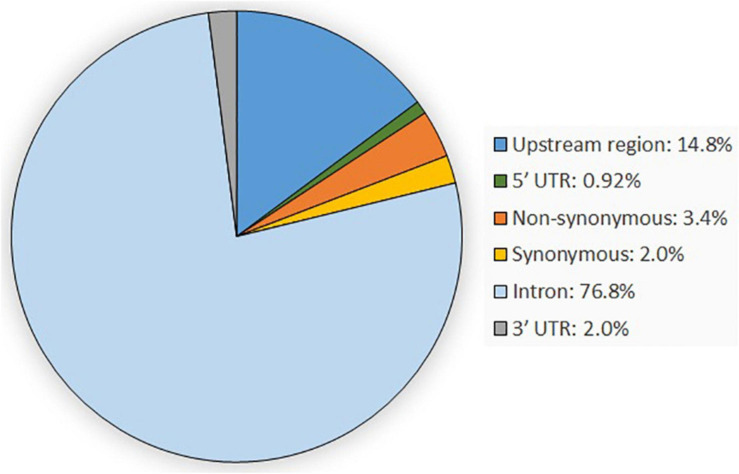
Overview of the genetic variant types occurring within the four casein genes *CSN1S1*, *CSN1S2*, *CSN2*, and *CSN3*.

In total, 647 SNPs were detected in the analysis of 80,685 bp obtained sequences within the casein gene regions of *CSN1S1*, *CSN2*, *CSN1S2*, and *CSN3* when compared with the goat reference sequence. Most of the detected variants were located in introns (76.82%), followed by variants in the upstream gene region (14.84%) and the non-synonymous variants (3.40%). The remaining SNPs were synonymous variants (2.01%) and variants located in the 3′-UTR (2.01%) and the 5′-UTR (0.92%) (Figure 1). SNP genotypes are available from the EVA under project ID: PRJEB42077 and can be found at https://www.ebi.ac.uk/ena/data/view/PRJEB42077.

#### CSN1S1

The reference sequence for *CSN1S1* (accession no. NC_030813) represents the alpha S1 casein variant *CSN1S1^∗^A* (XP_017904616), which includes the signal peptide. Sequence analysis of 22,807 bp revealed 226 SNPs with 9.9 SNPs per 1,000 sequenced base pairs. Among the identified SNPs, six were non-synonymous (Table 2), seven synonymous (Table 3), and 32 SNPs were located in the upstream region, four in the 3′-UTR, and 177 in introns (Supplementary Table 3).

**TABLE 2:** Allele frequency of non-synonymous variants in different goat breeds.

**Casein genes**	**CHR position^*a*^**	**SNP ID^*b*^**	**Allele**	**Exon (nt position)^*c*^**	**Amino acid**	**Amino acid position in whole protein^*d*^**	**Amino acid position in mature protein**	**Frequency of alternative allele**							
	
								**NU (*n* = 7)**	**D (*n* = 5)**	**NI (*n* = 7)**	**Tagg (*n* = 7)**	**SA (*n* = 2)**	**Bez ibex (*n* = 2)**	**Nu ibex (*n* = 2)**	**Alp ibex (*n* = 1)**
*CSN1S1*	6:85981710 6:85981711	rs268293069 rs655973384	CA/AT	3 (16/17)	His/Ile	23	8	0	0	0	0	0.25	0	0	0
	6:85982615	rs155505532	T/C	4 (8)	Leu/Pro	31	16	0.57	0.50	0.57	0.64	1.00	0	0	0
	6:85984154	ss7213522403	A/G	7 (4)	Ile/Val	59	44	0	0	0	0	0	0	0	0.50
	6:85987197	rs155505536	C/G	10 (21)	Gln/Glu	92	77	0.57	0.50	0.57	0.64	1.00	0.50	0	0
	6:85988705	rs268293072	G/A	12 (14)	Arg/Lys	115	100	0.57	0.50	0.57	0.64	0.75	0.50	0	0
*CSN2*	6:86015278	ss7213522526	G/C	1 (62)	Leu/Val	11	–	0	0	0	0	0	0	0.25	1.00
	6:86015259	ss7213522558	T/C	1 (43)	His/Arg	17	–	0	0	0	0	0	0	0.25	1.00
	6:86013169	rs652629715	T/G	2 (11)	Ile/Leu	49	–	0.07	0.10	0	0	0.25	0.50	0.25	1.00
	6:86008103	ss7213522487	G/A	7 (175)	Pro/Leu	198	148	0	0	0	0	0	0	0	1.00
	6:86008016	rs155505539	A/G	7 (89)	Val/Ala	227	177	0.50	0.50	0.50	0.57	0.57	0.50	0	1.00
*CSN1S2*	6:86079098	rs640625134	T/C	2 (23)	Phe/Ser	4	–	0	0	0	0.07	0.50	0	0	0
	6:86081790	ss7213522477	T/C	4 (16)	Phe/Ser	32	17	0	0	0	0	0	0	0.50	0
	6:86081887	ss7213522549	T/C	5 (2)	Ile/Thr	35	20	0	0	0	0	0	0	0.75	0
	6:86085160	rs659163710	G/C	11 (106)	Ala/Pro	134	119	1.00	0.90	1.00	1.00	1.00	1.00	1.00	1.00
	6:86085714	rs665830654	G/A	12 (7)	Glu/Lys	142	127	0.21	0.10	0	0	0	0	0.25	0
	6:86089407	ss7213522575	G/A	16 (11)	Ser/Asn	184	169	0	0	0	0	0	0	0.75	0
*CSN3*	6:86208927	ss7213522604	G/A	4 (70)	Ser/Asn	54	33	0	0	0	0	0	0	0	1.00
	6:86208939	ss7213522610	G/C	4 (82)	Ser/Thr	58	37	0	0	0	0	0	0	0	1.00
	6:86208960	rs268293109	A/G	4 (103)	Gln/Arg	65	44	0	0	0	0.07	0.50	0	0	0
	6:86209097	rs268293113	G/A	4 (240)	Asp/Asn	111	90	0.14	0.10	0	0	0	0	0	0
	6:86209263	rs651045868	T/C	4 (406)	Val/Ala	166	145	0.14	0.10	0	0	0	0	0	0

*NU, Nubian; D, Desert; NI, Nilotic; Tagg, Taggar; SA, Saanen; Bez ibex, Bezoar ibex; NU ibex, Nubian ibex; Alp ibex, Alpine ibex. –, The amino acid change did not occur in the mature protein sequence. ^*a*^*Capra hircus* autosome (CHR) position relative to reference sequences: accession no. NC_030813. ^*b*^rs, reference IDs for known SNPs are available from Ensembl (www.ensembl.org/); ss, new SNPs from our sequencing assigned by the European Variation Archive (EVA). ^*c*^nt, nucleotide position in the exon. ^*d*^Positions of the amino acids according to the reference protein sequence [*CSN1S1* (XP_017904616: 214 amino acids in the whole protein, 199 amino acids in the mature protein); *CSN2* (XP_005681778: 257 amino acids in the whole protein, 207 amino acids in the mature protein); *CSN1S2* (XP_013820127.2: 223 amino acids in the whole protein, 208 amino acids in the mature protein); *CSN3* (NP_001272516: 192 amino acids in the whole protein, 171 amino acids in the mature protein].*

**TABLE 3:** Allele frequency of synonymous variants in different goat breeds.

**Casein genes**	**CHR position^*a*^**	**SNP ID^*b*^**	**Allele**	**Exon (nt position)^*c*^**	**Amino acid**	**Amino acid position in whole protein^*d*^**	**Amino acid position in mature protein**	**Frequency of alternative allele**							
	
								**NU (*n* = 7)**	**D (*n* = 5)**	**NI (*n* = 7)**	**Tagg (*n* = 7)**	**SA (*n* = 2)**	**Bez ibex (*n* = 2)**	**Nu ibex (*n* = 2)**	**Alp ibex (*n* = 1)**
*CSN1S1*	6:85979976	ss7213522449	T/C	2 (26)	Leu	6	–	0	0	0	0	0	0	0.75	0.50
	6:85981703	rs672288350	T/A	3 (8)	Pro	20	5	0.36	0.40	0.43	0.36	0	0	0	0
	6:85982631	rs155505533	C/G	4 (24)	Leu	36	21	0.57	0.50	0.57	0.64	1.00	0	0	0
	6:85988712	ss7213522443	A/G	12 (21)	Lys	117	102	0.57	0.50	0.57	0.64	0.75	0.50	0	0
	6:85991559	ss7213522421	C/T	15 (24)	Asn	154	139	0.57	0.50	0.57	0.64	0.75	0.50	0	0
	6:85993377	ss7213522397	C/T	17 (51)	Tyr	180	165	0.57	0.50	0.57	0.64	0.75	0.50	0	0
	6:85993386	ss7213522438	A/G	17 (60)	Pro	183	168	0.07	0	0.14	0	0	0	0	0
*CSN2*	6:86015270	ss7213522504	C/T	1 (38)	Gln	13	–	0	0	0	0	0	0	0.25	1.00
*CSN3*	6:86206785	rs663488235	A/G	3 (26)	Gln	28	7	0	0.20	0.21	0.43	0.25	1.00	0	0
	6:86208859	rs155505563	C/T	4 (2)	Cys	31	10	0	0	0.07	0	0	0	0	0
	6:86208883	rs268293107	C/T	4 (26)	Phe	39	18	0	0	0.07	0.14	0	0	0	0
	6:86208889	ss7213522597	C/T	4 (32)	Asp	41	20	0	0	0	0	0	0	0.50	0
	6:86208958	rs268293108	T/C	4 (102)	Tyr	64	43	0.14	0.10	0	0.07	0.50	0	1.00	1.00

*NU, Nubian; D, Desert; NI, Nilotic; Tagg, Taggar; SA, Saanen; Bez ibex, Bezoar ibex; NU ibex, Nubian ibex; Alp ibex, Alpine ibex. –, The mutation occurred in the signal peptide. ^*a*^*Capra hircus* autosome (CHR) position relative to reference sequences (accession no. NC_030813). ^*b*^rs, reference IDs for known SNPs are available from Ensembl (www.ensembl.org/); ss, new SNPs from our sequencing assigned by the European Variation Archive (EVA). ^*c*^nt, nucleotide position in the exon. ^*d*^Positions of amino acids according to the reference protein sequence [*CSN1S1* (XP_017904616: 214 amino acids in the whole protein, 199 amino acids in the mature protein); *CSN2* (XP_005681778: 257 amino acids in the whole protein, 207 amino acids in the mature protein); *CSN1S2* (XP_013820127.2: 223 amino acids in the whole protein, 208 amino acids in the mature protein); *CSN3* (NP_001272516: 192 amino acids in the whole protein, 171 amino acids in the mature protein].*

Among the non-synonymous SNPs, one novel SNP was detected in wild Alpine ibex. This SNP was located at position CHR6:85984154 (ss7213522403, exon 7) and led to the amino acid substitution Ile44Val in the mature alpha S1 casein protein. The known non-synonymous SNPs rs155505536 and rs268293072 were found in all Sudanese breeds, Saanen goats, and Bezoar ibex; rs155505532 segregated in Sudanese breeds and Saanen, and the SNPs rs268293069 and rs655973384 were found in Saanen goats only (Table 2).

In the *CSN1S1* gene, five out of seven synonymous SNPs were novel [ss7213522449 (exon 2), ss7213522443 (exon 12), ss7213522421 (exon 15), ss7213522397 (exon 17), and ss7213522438 (exon 17)]. The SNPs ss7213522443, ss7213522421, ss7213522397, and ss7213522438 revealed synonymous mutations in the codons for the amino acids Lys_102_, Asn_139_, Tyr_165_, and Pro_168_ of the mature protein, respectively. The additional synonymous SNP ss7213522449 is located in the codon for the amino acid Leu_6_ in the signal peptide. Three out of the five novel synonymous SNPs (ss7213522443, ss7213522421, and ss7213522397) segregated in Sudanese breeds, Saanen, and Bezoar ibex (Table 3). The novel synonymous SNP ss7213522449 was identified in Nubian ibex and Alpine ibex, while the novel ss7213522438 SNP was found in Nubian and Nilotic goats only. The known synonymous SNP rs672288350 segregated in Sudanese breeds, and SNP rs155505533 was found in Sudanese breeds and Saanen goats (Table 3).

#### CSN2

The reference sequence for the *CSN2* gene (accession no. NC_030813) represents the beta casein variant *CSN2*^∗^C (XP_005681778), which includes the signal peptide. Sequencing of 15,071 bp revealed 109 SNPs with 7.23 SNPs per 1,000 sequenced base pairs. Among the identified SNPs, five were non-synonymous (Table 2), one synonymous (Table 3), and eight SNPs were located in the upstream region, two in the 3′-UTR, and 93 in introns (Supplementary Table 4). Three out of the five non-synonymous SNPs were novel. The novel SNPs ss7213522526 (exon 1), ss7213522558 (exon 1), and ss7213522487 (exon 7) (Table 2) led to the amino acid substitutions Leu11Val and His17Arg in the signal peptide and Pro148Leu in the mature protein of beta casein, respectively. All these novel SNPs were found in Alpine ibex, the first two ones also in Nubian ibex. The novel ss7213522487 SNP in exon 7 has a predicted deleterious effect on protein function (PROVEAN score = −4.947) using the PROVEAN tool (Supplementary Table 2). The known non-synonymous SNP rs652629715 segregated in most domesticated breeds (except Nilotic and Taggar) and all wild species, and SNP rs155505539 was found in all domesticated breeds and Bezoar and Alpine ibex.

In the *CSN2* gene, ss7213522504 (exon 1) was the only novel synonymous SNP in the codon Gln_13_ of the signal peptide. It was found in Nubian ibex and Alpine ibex.

#### CSN1S2

The reference sequence of the *CSN1S2* (accession no. NC_030813) represented the *CSN1S2^∗^A* variant of the alpha S2 casein protein (XP_013820127), which includes the signal peptide. On average, 8.5 SNPs were detected per 1,000 sequenced base pairs. In the sequence of 22,694 bp, 193 SNPs were found in comparison to the reference sequence. Among them, six were non-synonymous SNPs (Table 2), 38 were in the upstream region, four in the 5′-UTR, five in the 3′-UTR, and 140 in introns (Supplementary Table 5). In this study, three out of six non-synonymous SNPs were novel. The novel SNPs ss7213522477 (exon 4), ss7213522549 (exon 5), and ss7213522575 (exon 16) caused the amino acid substitutions Phe17Ser, Ile20Thr, and Ser169Asn in the mature alpha S2 casein protein, respectively (Table 2). All three novel SNPs were found in Nubian ibex. Among the known non-synonymous SNPs, rs640625134 was detected in Taggar and Saanen goats, the SNP rs659163710 was found in all domesticated breeds and wild species, and the SNP rs665830654 segregated in Nubian and Desert goats as well as in Nubian ibex (Table 2). Although all these three SNPs had entry ID numbers in the Ensembl database, none of them had been assigned to the *CSN1S2* gene. Therefore, we also provide here the annotated information of the SNPs rs640625134 (CHR6:86079098T > C, exon 2), rs659163710 (CHR6:86085160G > C, exon 11), and rs665830654 (CHR6:86085714G > A, exon 12), which lead to the amino acid substitutions of Phe4Ser in the signal peptide and Ala119Pro and Glu127Lys in the mature alpha S2 casein protein. No synonymous SNP was detected in the *CSN1S2* gene in this study.

#### CSN3

The reference sequence for *CSN3* (accession no. NC_030813) encodes the *CSN3*^∗^B kappa casein protein variant (NP_001272516), including the signal peptide. Sequencing of 20,113 bp of *CSN3* revealed 119 SNPs compared to the reference sequence with 5.9 SNPs per 1,000 sequenced base pairs. Among the identified SNPs, five were non-synonymous (Table 2), five synonymous (Table 3), and 20 were in the upstream region, two in 3′-UTR, and 87 in introns (Supplementary Table 6). Interestingly, all five non-synonymous and four out of the five synonymous SNPs reside in exon 4.

Two out of the five non-synonymous SNPs were novel. They were identified in Alpine ibex only (Table 2). The two novel non-synonymous SNPs ss7213522604 and ss7213522610 led to the amino acid substitutions Ser33Asn and Ser37Thr in the mature kappa casein protein, respectively. The known non-synonymous SNP rs268293109 was found in Taggar and Saanen goats and the SNPs rs268293113 and rs651045868 in Nubian and Desert goats.

In the *CSN3* gene, we also detected the novel synonymous SNP ss7213522597 (exon 4) in the codon for the amino acid Asp_20_ of the mature protein in Nubian ibex. The known synonymous SNP rs663488235 (exon 3) segregated in Desert, Nilotic, Taggar, and Saanen goats, as well as in Bezoar ibex, the SNP rs155505563 (exon 4) in Nilotic goats, the SNP rs268293107 in Nilotic and Taggar goats, and the SNP rs268293108 in Nubian, Desert, Taggar, and Saanen goats as well as in Nubian and Alpine ibex (Table 3).

### Casein Protein Variants

**FIGURE 2:**
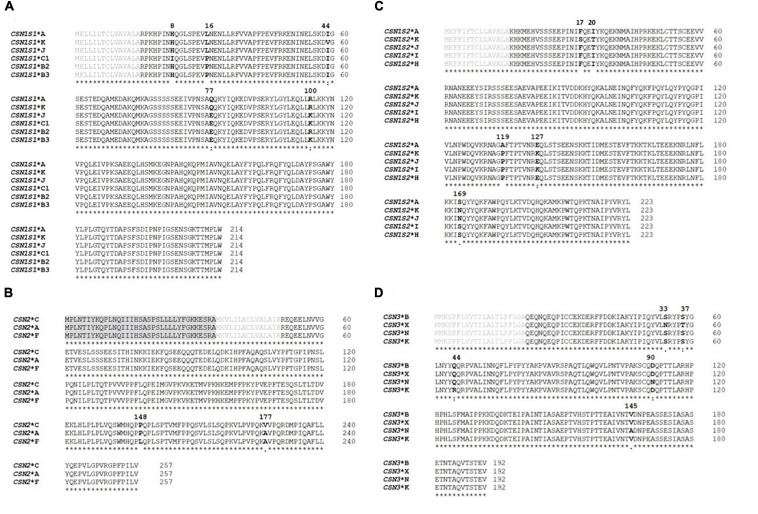
Amino acid sequence alignments of all casein protein variants detected in this study. **(A)** Alpha S1 casein, **(B)** beta casein, **(C)** alpha S2 casein, and **(D)** kappa casein. The reference protein variants were obtained from GenBank (*CSN1S1*: XP_017904616, 214 amino acids; *CSN2*: XP_005681778, 257 amino acids; *CSN1S2*: XP_013820127, 223 amino acids; *CSN3*: NP_001272516, 192 amino acids). The amino acid sequences for the casein variants were aligned and compared with the reference sequences using the multiple sequence alignment in Clustal Omega (http://www.ebi.ac.uk/Tools/msa/). The signal peptide sequences are labeled in *gray*, the mature protein sequences in *black*, and the amino acid differences are in *bold*. The positions of the amino acid substitutions in the mature protein are shown *above the sequence*. *Asterisk* indicates the same amino acids at the given position. *Colon* indicates conservation between groups of strongly similar properties—scoring >0.5 in the Gonnet PAM 250 matrix and both amino acids are similar to each other with respect to biological function. *Dot* indicates conservation between groups of weakly similar properties—scoring ≤0.5 in the Gonnet PAM 250 matrix.

The identified DNA sequence variants led to the recognition of 18 casein protein variants, including nine new ones: six protein variants in the alpha S1 casein, three in the beta casein, five in the alpha S2 casein, and four in the kappa casein (Figure 2). The frequency of the protein variants differed widely (Table 4).

**TABLE 4:** Milk protein variants and frequencies in different goat breeds.

**Locus**	**Allele**	**Allele frequency**							
		**Nubian (*n* = 7)**	**Desert (*n* = 5)**	**Nilotic (*n* = 7)**	**Taggar (*n* = 7)**	**Saanen (*n* = 2)**	**Bezoar ibex (*n* = 2)**	**Nubian ibex (*n* = 2)**	**Alpine ibex (*n* = 1)**
*CSN1S1*	A	0.43	0.50	0.43	0.36	–	0.50	1.00	0.50
	B2	–	–	–	–	0.25	–	–	–
	B3	0.57	0.50	0.57	0.64	0.50	–	–	–
	C1	–	–	–	–	0.25	–	–	–
	J	–	–	–	–	–	0.50	–	–
	K	–	–	–	–	–	–	–	0.50
*CSN2*	A	0.50	0.50	0.50	0.57	0.75	0.50	–	–
	C	0.50	0.50	0.50	0.43	0.25	0.50	1.00	–
	F	–	–	–	–	–	–	–	1.00
*CSN1S2*	A	–	0.10	–	–	–	–	–	–
	H	0.79	0.80	1.00	1.00	1.00	1.00	0.25	1.00
	I	0.21	0.10	–	–	–	–	–	–
	J	–	–	–	–	–	–	0.25	–
	K	–	–	–	–	–	–	0.50	–
*CSN3*	B	0.86	0.90	1.00	0.93	0.50	1.00	1.00	–
	K	–	–	–	0.07	0.50	–	–	–
	(M) N	0.14	0.10	–	–	–	–	–	–
	X	–	–	–		–	–	–	1.00

*Capra hircus* autosome (CHR) position relative to reference sequences (accession no. NC_030813): CSN1S1*A, CSN2*C, CSN1S2*A, and CSN3*B.

#### Alpha S1 Casein

Based on the DNA sequence information, we identified five amino acid substitutions. These contributed to the detection of six alpha S1 casein protein variants (Table 4). Among them, three protein variants were new and distinct from the *CSN1S1*^∗^A reference. A protein variant containing the amino acids Ile_8_, Pro_16_, Glu_77_, and Lys_100_ was similar to the protein variant *CSN1S1*^∗^C, with the only difference of threonine at position 195 instead of alanine. Therefore, this protein variant was named *CSN1S1*^∗^C1. *CSN1S1*^∗^C1 was found in Saanen goats. The protein variant containing the amino acids Glu_77_ and Lys_100_ was identified in Bezoar ibex only and was named *CSN1S1*^∗^J. The protein variant containing Val_44_ was observed in wild Alpine ibex and named *CSN1S1*^∗^K. The known protein variant *CSN1S1*^∗^A was found in Sudanese breeds, and in Bezoar, Nubian, and Alpine ibex, *CSN1S1*^∗^B2 in Saanen goats only, and *CSN1S1*^∗^B3 in Sudanese breeds and Saanen goats, but neither in Bezoar nor in Nubian or Alpine ibex. The *CSN1S1*^∗^B3 and *CSN1S1*^∗^C1 protein variants always occurred combined with the newly identified synonymous DNA variants G, T, and T in the positions CHR6:85988712, CHR6:85991559, and CHR6:85993377, respectively. In contrast, the same SNPs occurred with the alleles A, C, and C, respectively, in the *CSN1S1*^∗^A reference protein.

#### Beta Casein

The two known *CSN2*^∗^A and *CSN2*^∗^C variants and a new variant were found for beta casein. The new beta casein variant was detected in Alpine ibex only (Table 4). This new variant, which carried the amino acids Leu_148_ and Ala_177_, was named *CSN2*^∗^F. The *CSN2*^∗^F protein variant is always linked with allele T of the novel synonymous SNP at position CHR6:86015270 (ss7213522504), leading to the amino acid Gln_13_ in the signal peptide. The two known *CSN2*^∗^A and *CSN2*^∗^C variants were found in Sudanese breeds, Saanen goats, and Bezoar ibex. In addition, *CSN2*^∗^C was also found in Nubian ibex (Table 4).

#### Alpha S2 Casein

Five protein variants were found in the alpha S2 casein. Four of them are presented here for the first time (Table 4). The new protein variants are proposed to be named as *CSN1S2*^∗^H, *CSN1S2*^∗^I, *CSN1S2*^∗^J, and *CSN1S2*^∗^K according to the existing alphabetical order for this protein. The protein variant *CSN1S2*^∗^H contained the amino acid Pro_119_. This variant was found in all the examined breeds and ibex species. The protein variant *CSN1S2*^∗^I carried the amino acids Pro_119_ and Lys_127_. This variant was detected in Nubian and Desert goats. The protein variant *CSN1S2*^∗^J carried the amino acids Thr_20_, Pro_119_, and Asn_169_, and the *CSN1S2*^∗^K variant had the amino acids Ser_17_, Thr_20_, Pro_119_, Lys_127_, and Asn_169_. The two protein variants *CSN1S2*^∗^J and *CSN1S2*^∗^K were identified in Nubian ibex only. The *CSN1S2*^∗^A reference variant was detected in Desert goats only.

#### Kappa Casein

Four protein variants were found for kappa casein. One of them was new. This variant was identified in Alpine ibex. The new variant was most similar to the protein variant *CSN3*^∗^B, except for positions 33 and 37, where the new variant carried the amino acids asparagine and threonine, respectively. This variant was named as *CSN3*^∗^X (Table 4). The known variant *CSN3*^∗^B was fixed in Nilotic goats, Bezoar ibex, and Nubian ibex and was the most common variant in Nubian, Desert, Taggar, and Saanen, goats. The variant *CSN3*^∗^K was detected only in Taggar and Saanen goats. The variant *CSN3*^∗^N (as named in the new nomenclature by Gautam et al., 2019, but also called *CSN3*^∗^M in the study of Kiplagat et al., 2010) was found only in Nubian and Desert goats (Table 4).

## Discussion

Understanding the effects of different protein variants on human health and nutrition can be used for the selection and development of niche products.

The new alpha S1 casein variant *CSN1S1*^∗^J detected in Bezoar ibex has the amino acid substitutions Gln77Glu and Arg100Lys (compared to *CSN1S1*^∗^A). Since glutamine and glutamic acid are both polar, and arginine is similar to lysine (both contain long and flexible side chains with a positively charged end), we do not expect that the *CSN1S1*^∗^J variant has significantly different biochemical properties compared to the *CSN1S1*^∗^A variant; however, this expectation needs to be further investigated. Another new variant, *CSN1S1*^∗^K, detected in Alpine ibex has the amino acid substitution Iso44Val (compared to *CSN1S1*^∗^A). Both amino acids have large rigid aliphatic hydrophobic chains, and the biochemical properties of isoleucine and valine are similar. As such, major biochemical differences between the *CSN1S1*^∗^K and *CSN1S1*^∗^A variants are not expected. Interestingly, the protein variant *CSN1S1*^∗^B3 detected at high frequency in all Sudanese breeds and Saanen goats has been associated before with increased milk protein yield and high amounts of alpha S1 casein in milk. This, in turn, could alleviate the gross yield and quality of cheese production (Ambrosoli et al., 1988; Pirisi et al., 1994; Clark and Sherbon, 2000; Devold et al., 2011; Cebo et al., 2012).

For beta casein, two novel non-synonymous SNPs (ss7213522526 and ss7213522558) occurring in Nubian ibex and Alpine ibex were found in the signal peptide sequence. The mature protein is not affected by these SNPs. Because the encoded mature protein variant is not changed, no new variant name was assigned. Not assigning names to amino acid variants in the signal peptide, in the opinion of the authors, could lead to underestimating the role of the signal peptide. For example, the signal peptide changes might cause the protein to be mistargeted, leading to the protein not being excreted in the milk.

The beta casein protein variant *CSN2*^∗^A, which is believed to be the ancestral allele of *CSN2* (Chessa et al., 2005), was found in all examined domesticated goats breeds and Bezoar ibex with a frequency equal or above 0.5. The high frequency of *CSN2*^∗^A in domesticated goats has been described before in Saanen and Alpine goat breeds from France (Boulanger et al., 1984) and Italy (Marletta et al., 2005), as well as in goat breeds from India (Rout et al., 2010) and West Africa (Caroli et al., 2007). The *CSN2*^∗^A variant has been associated with high beta casein content in milk (about 5 g/L per allele) in comparison to *CSN2* null alleles (Roberts et al., 1992; Mahé and Grosclaude, 1993; Persuy et al., 1999; Neveu et al., 2002; Galliano et al., 2004; Cosenza et al., 2005; Caroli et al., 2006). Therefore, we hypothesize that the high frequency of the *CSN2*^∗^A variant in domesticated breeds could perhaps be the result from the selection of animals for milk with high protein and fat contents and good cheese-making properties (Tortorici et al., 2014; Vacca et al., 2014).

The *CSN2*^∗^A protein variant was not found in the wild Nubian and Alpine ibex. The absence of this variant in Nubian and Alpine ibex might be due to the low sample size in this study, but it could also be that the assumed ancestral allele is not the ancestral allele. Another hypothesis would be that the *CSN2*^∗^A variant has a fitness effect on large mountain goats. This, however, needs to be further investigated using a larger sample size of the Nubian and Alpine ibex, as well as looking into other mountain goat species. Another highly frequent protein variant is *CSN2*^∗^C, which was also found in all examined goat breeds, except in Alpine ibex. The high frequency of this protein variant was also evident in Northern and Southern Italian goat breeds (Chessa et al., 2005) and in Banat’s White Romanian goats (Kusza et al., 2016). The new beta casein protein variant *CSN2*^∗^F was detected in Alpine ibex only. Simulation shows that the substitution of proline to leucine at position 148 could lead to an enhanced cleavage of the protein by chymotrypsin.

For alpha S2 casein, besides the *CSN1S2*^∗^A protein variant, four new variants were detected in this study. For these new variants, preliminary names were suggested (*CSN1S2*^∗^H, *CSN1S2*^∗^I, *CSN1S2*^∗^J, and *CSN1S2*^∗^K). So far, 10 variants have been identified for the goat alpha S2 casein (see Table 1). However, only seven variants have been well characterized at the protein and DNA levels. Surprisingly, *CSN1S2*^∗^A was found in this study in Desert goats only. Since many other studies (Boulanger et al., 1984; Erhardt et al., 2002; Chiatti et al., 2005; Caroli et al., 2007) found this variant at high frequency, we had expected to find *CSN1S2*^∗^A in all goat breeds in our study.

With respect to kappa casein, *CSN3*^∗^B is not only the reference but also the most commonly found kappa casein variant in our study. This agrees with previous research (Kiplagat et al., 2010; Kupper et al., 2010; Strzelec and Niżnikowski, 2011). The *CSN3*^∗^K variant was detected in Taggar as well as in Saanen goats in our study, albeit it has not yet been reported before for Saanen goats from Europe (Kupper et al., 2010). The *CSN3*^∗^N variant, which was detected in Nubian and Desert goats, has been reported before at low frequency in the Small East Africa goat from Kenya and Long Eared Somali goats from Ethiopia and Somalia (Kiplagat et al., 2010). The new variant *CSN3*^∗^X that was detected in Alpine ibex was similar to the protein variant *CSN3*^∗^B, except for positions 33 and 37, where *CSN3*^∗^X carried the amino acids asparagine and threonine, respectively. Concerning the amino acid substitutions Ser33Asp and Ser37Thr, asparagine has similar biochemical properties to serine, while threonine at position 37 might enhance cleavage of the protein by proteinase K. The isoelectric focusing (IEF) pattern of the new variant *CSN3*^∗^X was not experimentally tested, but it was predicted using ExPASy. The predicted IEF was 5.53. If true, *CSN3*^∗^X belongs to the B^*IEF*^ group, while *CSN3*^∗^B was classified in the A^*IEF*^ group. Since the B^*IEF*^ group is favorable for improving milk protein content and cheese-making properties, the new variant is an interesting target for milk and cheese production.

Most of the novel protein variants detected in our study were found in Nubian and Alpine ibex. This underlines the necessity to pay more attention to the study and conservation of endangered species in order to protect valuable genetic resources.

## Conclusion

In this study, novel genetic variations of goat casein genes were discovered by capture sequencing. Most of the genetic variations, especially the non-synonymous polymorphisms, were identified in the critically endangered Nubian ibex and Alpine ibex. Therefore, we would like to emphasize and highlight the importance of preservation and studying rare and endangered species. It is noteworthy that nine new protein variants were found for the first time in the DNA sequences of the casein genes. Three protein variants in the *CSN1S1* gene were identified in Saanen goats, Bezoar ibex, and Alpine ibex. In the *CSN2* and *CSN3* genes, one additional protein variant was detected in Alpine ibex in each gene. Four new protein variants that were found in the *CSN1S2* gene occurred in all studied goat breeds and species. The identified novel protein variants are of interest not only for their effect on protein and milk composition but also for evolutionary studies on milk protein genes. Unfortunately, neither RNA nor milk samples of the studied goat breeds were available. Therefore, further investigation is necessary to examine the expression of the nine new variants on the protein level to validate and confirm the outcomes of this study.

## Data Availability Statement

The original contributions presented in the study are included in the article/Supplementary Material. SNP genotypes are available from the European Variant Archive (EVA) under project ID: PRJEB42077, and can be found at https://www.ebi.ac.uk/ena/data/view/PRJEB42077. The DNA sequencing dataset is available at the NCBI Short Read Archive under BioProject ID: PRJNA683771. The dataset in this study can be found at http://www.ncbi.nlm.nih.gov/bioproject/683771. Further inquiries can be directed to the corresponding authors.

## Ethics Statement

All samples were collected with permission from the owners of the animals and according to the animal protection law in Sudan. Written informed consent was obtained from the owners for the participation of their animals in this study.

## Author Contributions

SR, DA, and GB conceived and designed the study. SR, MR, and LH provided the samples. SR, MR, and SK performed the experiments. SR and DA analyzed the data. SR interpreted the data and drafted the manuscript. DA and GB helped to draft the manuscript. AS, LH, MR, and SK did critical revision of the manuscript. All authors read and approved the final manuscript.

## Conflict of Interest

The authors declare that the research was conducted in the absence of any commercial or financial relationships that could be construed as a potential conflict of interest.

## Footnotes

1 https://www.ncbi.nlm.nih.gov/

2 http://provean.jcvi.org

3 http://web.expasy.org

## Supplementary Material

Supplementary Figure 1 The geographical location of the Sudanese samples.

Supplementary Figure 2 The average read depth across all SNPs.

Supplementary Table 1 Characterization of the samples by species, breed, origin, and number of sequenced animals.

Supplementary Table 2 PROVEAN predictions of novel non-synonymous SNPs.

Supplementary Table 3 Sequence variation at the *CSN1S1* gene.

Supplementary Table 4 Sequence variation at the *CSN2* gene.

Supplementary Table 5 Sequence variation at the *CSN1S2* gene.

Supplementary Table 6 Sequence variation at the *CSN3* gene.
