# Supplementary figures and images for: Capture Sequencing to Explore and Map Rare Casein Variants in Goats
Source: Front Genet. 2021 Feb 23;12:620253. doi: 10.3389/fgene.2021.620253 (PMC7940697; doi:10.3389/fgene.2021.620253)

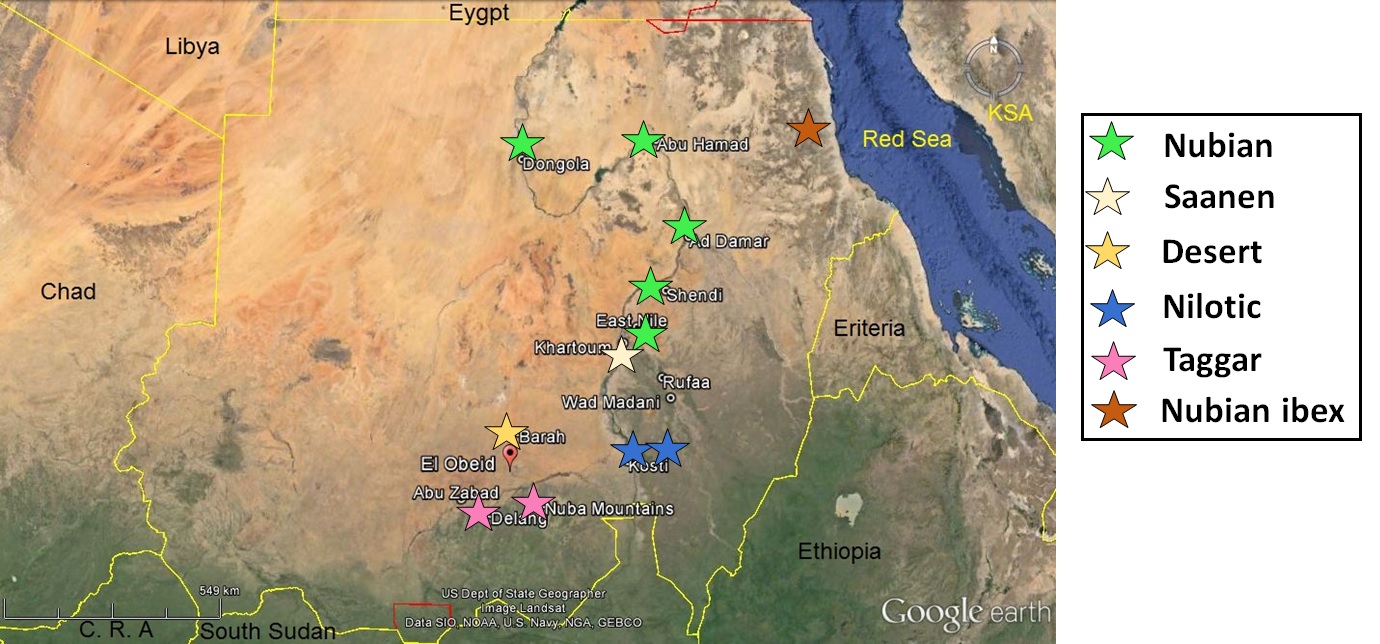

Supplement: Supplementary Figure 1 — The geographical location of the Sudanese samples. [file Image_1.JPEG]

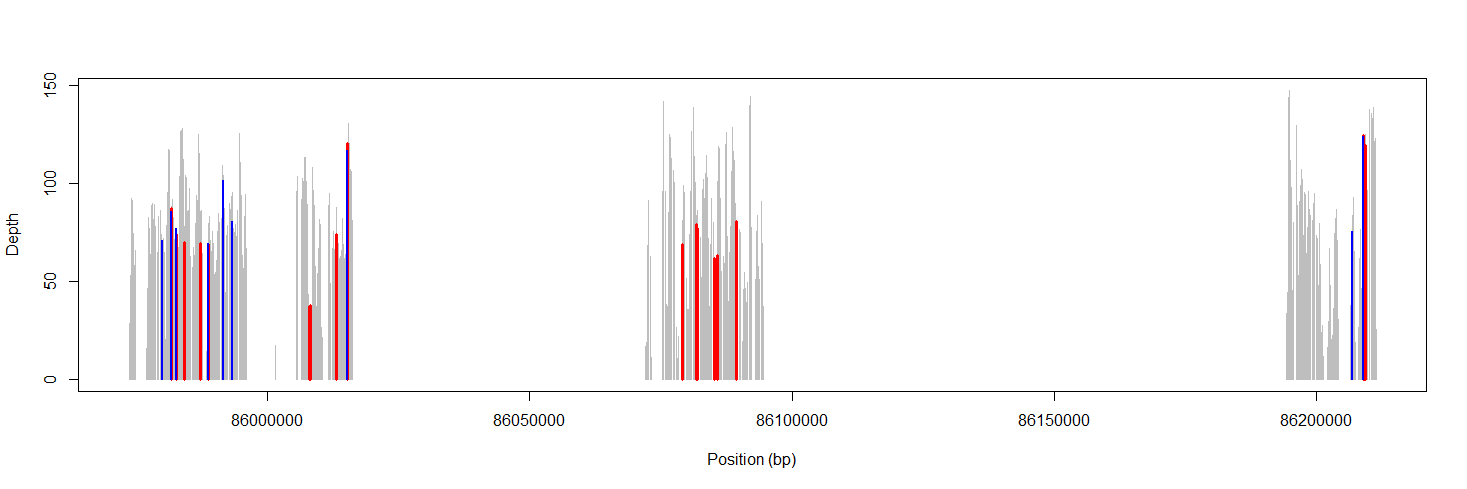

Supplement: Supplementary Figure 2 — The average read depth across all SNPs. [file Image_2.PNG]
