# Supplementary material for: Capture Sequencing to Explore and Map Rare Casein Variants in Goats
Source: Front Genet. 2021 Feb 23;12:620253. doi: 10.3389/fgene.2021.620253 (PMC7940697; doi:10.3389/fgene.2021.620253)
Supplement: Supplementary Table 1 — Characterization of the samples by species, breed, origin, and number of sequenced animals. [file Table_1.docx]

**Supplementary Table 1:** Characterization of the samples by species, breed, origin, and number of sequenced animals

| **Species** | **Breed** | **Origin** | **Number of animal** |
| --- | --- | --- | --- |
| *Capra hircus* | Nubian | Sudan | 7 |
| *Capra hircus* | Desert | Sudan | 5 |
| *Capra hircus* | Nilotic | Sudan | 7 |
| *Capra hircus* | Taggar | Sudan | 7 |
| *Capra hircus* | Saanen | Sudan (Netherland) | 2 |
| *Capra aegagrus aegagrus* | Bezoar ibex | Germany | 2 |
| *Capra nubiana* | Nubian ibex | Sudan | 2 |
| *Capra ibex* | Alpine ibex | Germany | 1 |
